# Fungal bioassays for environmental monitoring

**DOI:** 10.3389/fbioe.2022.954579

**Published:** 2022-08-25

**Authors:** Douglas M. M. Soares, Dielle P. Procópio, Caio K. Zamuner, Bianca B. Nóbrega, Monalisa R. Bettim, Gustavo de Rezende, Pedro M. Lopes, Arthur B. D. Pereira, Etelvino J. H. Bechara, Anderson G. Oliveira, Renato S. Freire, Cassius V. Stevani

**Affiliations:** ^1^ Departamento de Química Fundamental, Instituto de Química, Universidade de São Paulo, São Paulo, Brazil; ^2^ Research Centre for Greenhouse Gas Innovation (RGCI-POLI-USP), University of São Paulo, São Paulo, Brazil; ^3^ Departamento de Bioquímica, Instituto de Química, Universidade de São Paulo, São Paulo, Brazil; ^4^ Department of Chemistry and Biochemistry, Yeshiva University, New York, NY, United States

**Keywords:** ascomycete, basidiomycete, bioluminescence, ecotoxicology, metal cation, organic pollutant

## Abstract

Environmental pollutants are today a major concern and an intensely discussed topic on the global agenda for sustainable development. They include a wide range of organic compounds, such as pharmaceutical waste, pesticides, plastics, and volatile organic compounds that can be found in air, soil, water bodies, sewage, and industrial wastewater. In addition to impacting fauna, flora, and fungi, skin absorption, inhalation, and ingestion of some pollutants can also negatively affect human health. Fungi play a crucial role in the decomposition and cycle of natural and synthetic substances. They exhibit a variety of growth, metabolic, morphological, and reproductive strategies and can be found in association with animals, plants, algae, and cyanobacteria. There are fungal strains that occur naturally in soil, sediment, and water that have inherent abilities to survive with contaminants, making the organism important for bioassay applications. In this context, we reviewed the applications of fungal-based bioassays as a versatile tool for environmental monitoring.

## Introduction

The expansion of the anthropogenic activities to attend to the rising global demand for a diversity of products has generated an exponential increase in the release of different pollutants into the air, soil, and aquatic compartments (Esposito et al., 2021). These pollutants, that fall into a broad category of xenobiotic compounds, are released in large quantities into the environment, very often on the fringes of the law, and, unfortunately, they are not readily degraded by indigenous microfauna and flora, being bioaccumulate and biomagnified along the food chain (Wasi et al., 2013; Zenker et al., 2014). Among the ubiquitous environmental pollutants, phenolic compounds, and transition metal cations represent some of the major toxicants present in surface water (Wasi et al., 2008, 2013; Tabrez and Ahmad, 2011).

Thereby, these pollutants change the balance of biodiversity and have led to serious health problems for humans and other living organisms (Alharbi et al., 2018; Maurya and Pachauri, 2022). Monitoring pollution levels is essential for restoration (bio)remediation, as well as it might provide the basis for other pollutants control management practices (Garg et al., 2022). There are three classes for environmental pollution monitoring: physical (such as odor, color, taste, porosity, temperature, conductivity, and aggregate stability), chemical (including parameters such as redox potential, salinity, and biological and chemical oxygen demand), and biological (microbes, plants, and animals) indicators (Maurya and Pachauri, 2022). Among these classes, bioassays using biological indicators have been extensively used to either analyze target chemicals in water, sediment, and soil samples and for evaluating the relative ecotoxicological impact of such chemicals in these matrices (Viegas, 2021). Mainly due to their sensitivity and reproducibility, bioassays have great advantages over other physical or chemical methods to detect pollutants (Maurya and Pachauri, 2022). Given their morphological and ecological diversity, exhibiting from unicellular to multicellular forms with different growth rates (Chethana et al., 2021; Hurdeal et al., 2021), besides their tolerance and ability to survive in contaminated sites (Rani et al., 2014; Zapana-Huarache et al., 2020; Hamad et al., 2021), fungi are considered promising candidates as biological indicators in bioassays.

**FIGURE 1:**
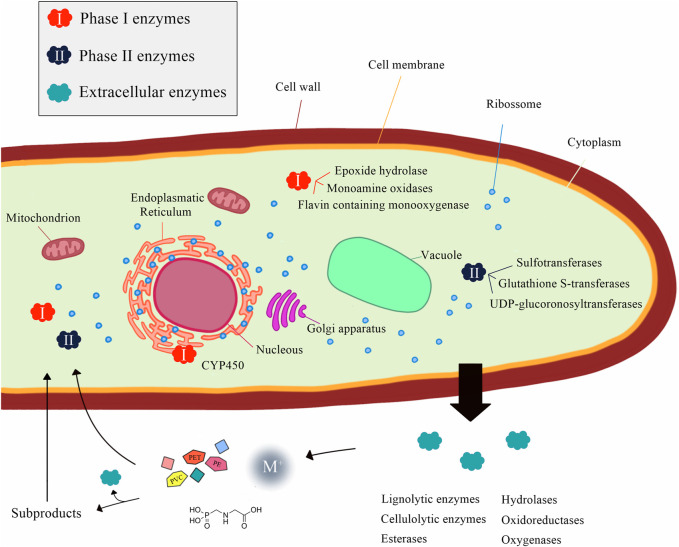
Intra- and extracellular enzymatic systems in fungi related to the biodegradation of environmental pollutants.

Moreover, fungi possess an extraordinary repertoire of enzymes that makes them able to degrade a wide range of environmental pollutants, even those with complex structures, by combining different mechanisms from their intracellular and extracellular enzymatic systems (Sánchez, 2020; Naveen et al., 2022) (Figure 1). The intracellular enzymatic system, including Phase I (involved in oxidation, as cytochrome P450 family epoxidase) and Phase II enzymes (related to conjugation processes, such as transferases), serves as a detoxifying mechanism and plays an important role in fungal adaptability (Shin et al., 2018; Sánchez, 2020). Additionally, the extracellular enzymatic system, which includes hydrolases (such as the well-known cutinolytic enzymes, able to hydrolyze cuticular polymers even without undergoing interfacial activation) and nonspecific oxidoreductases, comprising the class II peroxidases, laccases, and unspecific peroxygenases, accept a wide range of substrates, acting in the degradation of complex structures and favoring their uptake by the cell (Sánchez, 2020).

A great diversity and combination of these enzymatic complexes can be found in several fungal species as, for instance, some wood-degrading basidiomycetes, which produce lignin peroxidases, manganese peroxidases, and laccases, along with oxygenases, oxidases/dehydrogenases, and cellulolytic enzymes (Schwartz et al., 2018; Naveen et al., 2022). In this scenario, the comprehension of the molecular pathways used for fungi exposed to different environmental pollutants could expand their implementation in bioassays for the detection and management of contaminated areas (Sánchez, 2020; Naveen et al., 2022). Herein, we review the application of yeasts, other ascomycetes, and basidiomycetes in ecotoxicological studies to predict the toxicity of environmental pollutants.

### Fungal bioassays for environmental monitoring

Fungi, together with bacteria, microalga, and protozoa, are among the frontline biota exposed to environmental pollutants, which makes them an interesting model to be exploited in ecotoxicological assays (Viegas, 2021). Indeed, several fungal species have been employed in bioassays for environmental pollutants monitoring (Table 1).

**TABLE 1:** Some examples of bioassays for environmental pollutants using fungal species.

Environmental pollutant	Organism	References
polystyrene nanoparticles	*Anguillospora crassa, Tetracladium marchalianum, Tetrachaetum elegans, Articulopora tetracladia,* and *Tricladium spendens*	Seena et al. (2019)
2,4,6-trichlorophenol, 4-cyanophenol, 4-nitrophenol, phenol, 4-chlorophenol, 4-methoxyphenol	*Gerronema viridilucens*	Ventura et al. (2020)
Cd, Cu(II), phenol, 4-nitrophenol	*Neonothopanus gardneri*	Ventura et al. (2021)
Cd, Ni, Cu, Zn, Cr, and Pb	*Rhizopus* sp., *Cladosporium* sp., *Penicillium* sp., *Curvularia* sp., *Fusarium* sp., *Alternaria* sp., *Pestalotiopsis* sp., *Aspergillus* sp., *Trichoderma* sp	Mahanty et al. (2021)
polyethylene leachates, polyethylene terephthalate leachates, and polypropylene leachates	*Fusarium oxysporum* and *Phanerochaete chrysosporum*	Li et al. (2022)

**FIGURE 2:**
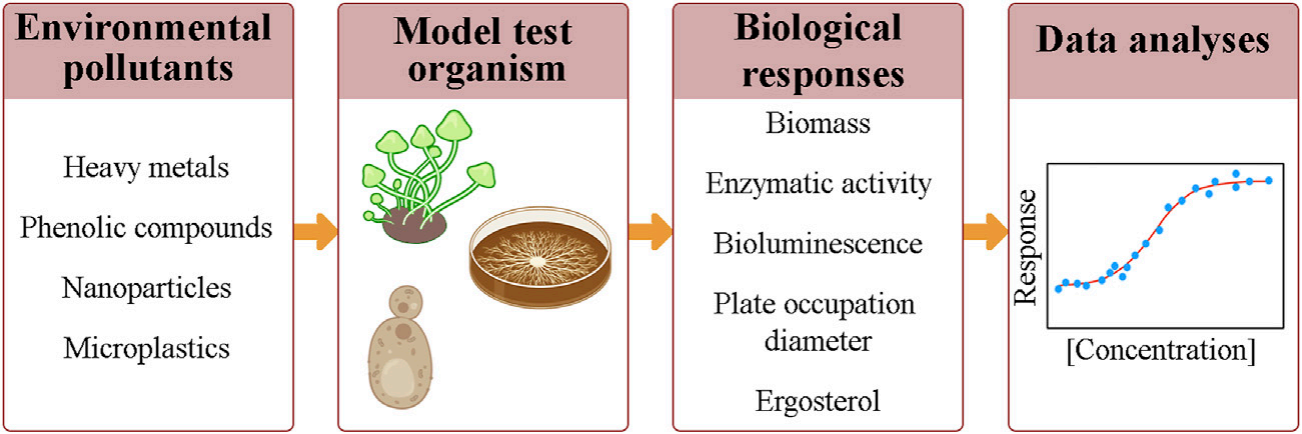
Common steps in ecotoxicological assays, which include: the exposure of a model test organism to different concentrations of a chemical compound or dilutions of an environmental sample; the monitoring of biological responses; and the obtention of a concentration-response curve.

Most reports regarding the use of fungi in bioassays show the effect of soil pollutants or pure substances, including metal cations, organochlorine, and phenolic compounds, on the growth of mycelium cultures using methodologies based on biomass quantification, enzymatic activity, bioluminescence, and plate occupation diameters (Mendes and Stevani, 2010; Chan-Cheng et al., 2020; Ventura et al., 2020) (Figure 2).

Recently, the application of fungi bioluminescence system has been demonstrated to respond, albeit not specifically, to several metal cations such as Cd(II) and Cu(II), and phenolic compounds by bioluminescent basidiomycetes *Neonothopanus gardneri, Gerronema viridilucens*, *Armillaria mellea,* and *Mycena citricolor* (Weitz et al., 2002; Mendes et al., 2010; Mendes and Stevani, 2010; Stevani et al., 2013; Ventura et al., 2020, 2021). In an agar medium, the bioassay with the fungus *N. gardneri*, which responds in a more sensitive and repeatable way, can be conducted by exposing the mycelium for 30 min or 24 h to an aqueous solution containing the toxicant. Bioluminescence is a precocious endpoint that can indicate the toxic effect of the sample. There is evidence that the decrease of light emission in the presence of the toxicant is related to the uncoupling of mitochondrial oxidative phosphorylation, leading to the impairment of ATP biosynthesis (Ventura et al., 2020). Likewise, other bioluminescent microorganisms, like bacteria, respond to toxic substances by decreasing light emission proportionally to concentration of the toxicant (Hollis et al., 2000; Weitz et al., 2002; Mendes and Stevani, 2010; Ventura et al., 2020, 2021).

Besides detecting air and soil pollutants, different fungal species have also been employed as water bioindicators to sense the quality of the environment (Maurya and Pachauri, 2022). Despite fungi respond to environmental pollution, bioassays using aquatic fungi are scarce, even though they would better mirror the impact of hazardous pollutants in freshwater ecosystems (Ortiz-Vera et al., 2018). The main issue with the use of aquatic fungi in bioassays is the challenge to determine work experimental conditions that are close to the natural ones (Ittner et al., 2018). There are some reports on the use of classical growth-based methodologies, but with fungi isolated from polluted waters (Duddridge and Wainwright, 1980; Chandrashekar and Kaveriappa, 1989; Jaeckel et al., 2005). Nevertheless, it is more common to find metagenomic studies from aquatic fungi instead of ecotoxicological bioassays, once this tool allows the assessment of the impact of a toxic agent in a broader way at the community level (Selvarajan et al., 2019; Usharani, 2019; Ogwugwa et al., 2021; Zhang et al., 2022).

Usually, the fungal communities are evaluated by the quantification of ergosterol, an alternative way to measure growth (Bundschuh et al., 2011; Dimitrov et al., 2014; Zubrod et al., 2015; Donnadieu et al., 2016; Gessner, 2020). Ergosterol is a major membrane component on fungi, with similar functions to cholesterol in animal cells but being absent in animal and plant membranes. Hence, its quantification is a useful method because the mycelium is not easily separated from the leaf tissue and, therefore, an analysis method focusing on specific fungal cell constituents, like chitin or ergosterol, is required for this type of bioassay (Gessner, 2020; Baudy et al., 2021). Additionally, leaf mass loss can be used to measure fungal biomass in aquatic hyphomycetes, a group of saprotrophic fungi adapted to submerged leaf litter (Baudy et al., 2021). Generally, there is a strong linear association between leaf mass loss and the cumulative activity of fungal enzymes (Flores et al., 2014; Baudy et al., 2021).

Due to their high sensitivity to air pollutants, such as nitrogen oxides (NOx) or SO_2_, fungal associations with roots (mycorrhiza) or algae (lichens) are considered good indicators of air quality (Hage-Ahmed et al., 2019; Castro e Silva et al., 2020; Anderson et al., 2022). Considering that fungal-based air pollution bioassays are scarce, this property becomes promising for developing bioassays for the detection of atmospheric pollutants based on fungal associations. Indeed, most recent studies have focused on correlating air pollution with sporulation or biodiversity of fungal communities present in particulate matter, usually comparing fungal and bacterial diversity by DNA sequencing (Du et al., 2018; Fan et al., 2019; Stevens et al., 2021). However, evidence suggests that spores concentration in air is more affected by meteorological factors than by pollution, a finding that is difficult to confirm due to the strong relationship between air pollution and meteorological factors (Pyrri and Kapsanaki-Gotsi, 2017).

Among fungi, the budding yeast *Saccharomyces cerevisiae* is considered a relevant animal-alternative tool in ecotoxicological studies, mainly due to its non-pathogenicity, experimental amenability, cost-effectiveness, and the public availability of genomics and bioinformatics resources which aid the interpretation of its biological processes (Viegas, 2021). For instance, the exposure of an engineered strain of *Saccharomyces cerevisiae* expressing the firefly luciferase gene (*luc*) from *Photinus pyralis* to herbicides like mecoprop, diuron, and the Cu ions interferes with cell membrane integrity. This damage leads to defense responses to neutralize the toxic agent that consumes ATP and compete with the ATP-dependent bioluminescence, which ultimately decreases light emission (Martin-Yken, 2020).

## Conclusion and perspectives

Fungi are multi or unicellular organisms that have been widely applied as a biological indicator responding to several toxicants. Several reports have shown the efficient use of fungi to assess the toxicity of metals, organic compounds, and inorganic contaminants in bioassays using the inhibition of enzymatic activity, mycelium growth, ergosterol measurement, and bioluminescence intensity as parameters. Additionally, the current genetic engineering approaches in yeasts, such as *S. cerevisiae*, make possible the expression of optimized-recombinant proteins which, combined with a specific detection system, can expand both the type and the intensity of the response signal (Peltola et al., 2005; Matsuura et al., 2013; Li et al., 2016; Sun and Wang, 2021). Finally, the efficient use of modern techniques of molecular biology such as genome sequencing, heterologous expression of enzymes, the transformation of filamentous fungi, and genetic modifications using the CRISPR/Cas system (Li et al., 2017; Song et al., 2019) can harness tools to simplify the construction of new fungal sensors for more accurate bioassays.
